# Quantitative Assessment of Motor Function for Patients with a Stroke by an End-Effector Upper Limb Rehabilitation Robot

**DOI:** 10.1155/2020/5425741

**Published:** 2020-04-08

**Authors:** Yali Liu, Qiuzhi Song, Chong Li, Xinyu Guan, Linhong Ji

**Affiliations:** ^1^Department of Mechanical Engineering, Beijing Institute of Technology, Haidian, Beijing, China; ^2^Division of Intelligent and Biomechanical System, State Key Laboratory of Tribology, Tsinghua University, Haidian, Beijing, China

## Abstract

With the popularization of rehabilitation robots, it is necessary to develop quantitative motor function assessment methods for patients with a stroke. To make the assessment equipment easier to use in clinics and combine the assessment methods with the rehabilitation training process, this paper proposes an anthropomorphic rehabilitation robot based on the basic movement patterns of the upper limb, point-to-point reaching and circle drawing movement. This paper analyzes patients' movement characteristics in aspects of movement range, movement accuracy, and movement smoothness and the output force characteristics by involving 8 patients. Besides, a quantitative assessment method is also proposed based on multivariate fitting methods. It can be concluded that the area of the real trajectory and movement accuracy during circle drawing movement as well as the ratio of force along the sagittal axis in backward point-to-point movement are the unique parameters that are different remarkably between stroke patients and healthy subjects. The fitting function has a high goodness of fit with the Fugl-Meyer scores for the upper limb (*R*^2^ = 0.91, *p* = 0.015), which demonstrates that the fitting function can be used to assess patients' upper limb movement function. The indicators are recorded during training movement, and the fitting function can calculate the scores immediately, which makes the functional assessment quantitative and timely. Combining the training process and assessment, the quantitative assessment method will farther expand the application of rehabilitation robots.

## 1. Introduction

Stroke is one common disease caused by abnormal blood supply, about 15% hemorrhagic and 85% ischemic blood [1]. Almost 85% of patients with a stroke have difficulties with their hemiplegic upper limb during daily life [2]. Once a stroke patient has been in steady state after drug therapy, he/she will receive many rehabilitation trainings to promote recovery and prevent complications [3]. To be clear about the motor function state of patients and make customized training schedules for patients, therapies should make function assessment.

In the early stage, researchers focused on muscle strength assessment [4]. With the increasing cases of stroke patients, therapists and researchers had developed many function assessment scales such as Brunnstrom Scales and Fugl-Meyer Scales [5–7]. However, with rehabilitation training methods changed by intelligent equipment such as rehabilitation robots, the assessment methods are needed to be improved. Scales' assessment methods are dependent on therapists' experience. To make the assessment methods impersonal and combined with intelligent equipment, it is necessary to develop quantitative methods for motor function assessment.

Many researchers have made studies on quantitative assessment methods. Ellis et al. [8] and Murphy et al. [9] analyzed the range of the joint angle and movement during point-to-point reaching movement by an optoelectronic three-dimensional motion capture system and pointed out that the absolute range of movement was usually influenced by individual difference. Fasoli et al. [10] proposed that the movement time and peak velocity can be used to analyze the effect of instructions on functional performance for patients. Murphy et al. [11] and Wagner et al. [12] analyzed the average velocity, the maximum velocity, and peak velocity during the drinking daily activity and found the maximum velocity was different between different stages of patients. What is more, researchers analyzed the movement accuracy by the movement direction deviation, the movement straightness [13], and ellipticity [14], which was found to have remarkable relevance with the impairment degree for stroke patients. Researchers also analyzed the movement smoothness by calculating the number of peak velocity, but there was some inconsistency in the relationship between it and patients' recovery stages. In 2006, Kahn et al. [13] found the increased number reflexed improvement during rehabilitation programs for chronic patients. However, Colombo et al. [15] and Panerese et al. [16] found the number reduced during patients' improvement.

Although many researches have been made on the quantitative assessment, inconsistency still exists in the relationship between the parameters and the motor function, which may be caused by the different movement patterns in the researches. Some researchers analyzed the fetching for the glass movement in daily life [13, 17–19], and some researchers analyzed the circle drawing movement in the desired trajectory [14, 15]. It is important to analyze the principal movement patterns for patients' motor function assessment. Besides, most of the quantitative assessment methods relied on the complicated optoelectronic three-dimensional motion capture system, which may be difficult to use in clinics [20–22]. It is also important to make quantitative assessment equipment easier to establish for clinical use.

Intending to make the movement patterns during function assessment standard and make the quantitative assessment equipment easier to use, this paper analyzes the principal movement patterns and proposes an anthropomorphic rehabilitation robot based on the principal movement patterns. Besides, this paper analyzes the patients' movement characteristics in aspects of movement range, movement accuracy, movement smoothness, and output force characteristics during the principal movement patterns. It also proposes the quantitative functional assessment method based on the parameters, which were remarkably correlated with functional assessment scores. This proposed assessment method based on the rehabilitation robot combines the assessment process with the rehabilitation training process, which will further the automation of rehabilitation robots and the application of rehabilitation robots in clinics.

## 2. Materials and Methods

### 2.1. The Movement Patterns of Upper Limb for Motor Function Assessment

The point-to-point reaching movement was considered the basic movement pattern of the upper limb that made up most of the daily behavior [23]. The point-to-point reaching movement primarily involved shoulder flexion/extension and elbow flexion/extension. In order to assess more coordinate movements between shoulder and elbow joints, the circle drawing movement was added as the second basic movement pattern, which involved more ranges of shoulder internal/external rotation and shoulder abduction/adduction as well as shoulder flexion/extension and elbow flexion/extension [14].

**Figure 1:**
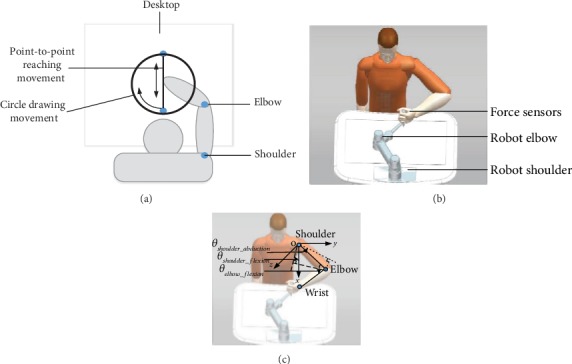
The movement patterns of upper limb and the structure of end-effector upper limb rehabilitation robot for assessment (EEULRbot): (a) the movement patterns of upper limb, (b) the structure of EEULRbot with human model, and (c) the definition of angles of shoulder and elbow joints.

To demonstrate the circle drawing movement involved more coordinate movement between shoulder and elbow joints, nine healthy people were involved to act the required movement patterns. Every person was required to perform a point-to-point reaching movement and a circle drawing movement, whose diameter was the range of point-to-point reaching movement in a plane, shown in Figure 1(a).

The movement of the shoulder and elbow joints was recorded based on infrared reflective markers attached to the human skeleton according to Plug-in-gait by VICON motion capture (Oxford Metrics Ltd, Oxford, UK) with 8 digital cameras. The inverse kinematics and dynamics were used to calculate the movement angle and the involved muscle activation in AnyBody Modeling System (AMS) (version 5.3, AnyBody Technology, Denmark).

The motion coordination was evaluated by the cocontraction coefficient (CC) of muscles, which was calculated according to [24].

(1) $$\begin{array}{c} \text{CC}=\frac{\int_{t_{1}}^{t_{2}}\min\left(A_{\text{agonist}},A_{\text{antagonist}}\right)}{\int_{0}^{t}\left(A_{\text{agonist}}+A_{\text{antagonist}}\right)}. \end{array}$$

*A*_agonist_ and *A*_antagonist_, respectively, represented the muscle activation of agonist and antagonist, and min(*A*_agonist_, *A*_antagonist_) represented the smaller one between the agonist and antagonist muscle activation. The integrals of ∫_*t*_1__^*t*_2_^min(*A*_agonist_, *A*_antagonist_) meant the integrated myoelectricity (IEMG) which is calculated by the time integral of the smaller one between the agonist and antagonist muscle activation. ∫_0_^*t*^(*A*_agonist_ + *A*_antagonist_) meant the total integrated myoelectricity (IEMG) which is calculated by the time integral of the sum of the agonist and antagonist muscle activation. *t*_1_ and *t*_2_ represented the start and the final time of the overlapping of the agonist and antagonist muscle activation during one movement.  *t* represented the entire time of one movement.

Primary agonist and antagonist for shoulder and elbow joints are shown in Table 1 [25].

**Figure 2:**
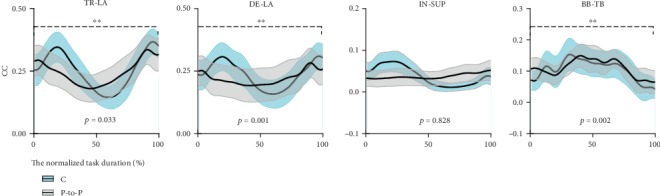
The cocontraction coefficient of different muscles during circle drawing and point-to-point reaching movement.

As shown in Figure 2, the co-contraction coefficients (CC) between TR-LA, DE-LA, and BB-TB were bigger in circle drawing movement than those in point-to-point reaching movement, which demonstrated that circle drawing movement pattern involved more muscle co-contraction than point-to-point reaching movement in shoulder flexion/extension, shoulder abduction/adduction, and elbow flexion/extension movement.

The basic movement for upper limb motor function assessment should include two movement patterns: one was point-to-point reaching movement that made up most of human behavior in daily life and the other one was the circle drawing that involved more coordinate movements between shoulder and elbow joints.

TR, LA, DE, LA, IN, SUP, BB, and TB had the same meaning as those in Table 1. C represented the circle drawing movement and P-to-P represented the point-to-point reaching movement. The *x*-axis represented the normalized action cycle percentage. 100% in *x*-axis meant the entire action, which was the forward and backward line drawing for P-to-P movement and the clockwise circle round drawing for circle drawing movement. ∗∗ described that the difference between circle drawing and point-to-point reaching was in statistical significance (*p* < 0.05).

### 2.2. Design of the Upper Limb Rehabilitation Robot for Assessment

The upper limb rehabilitation robot for assessment should have less intervention to patients' behavior and record patients' basic movement characteristics. We designed an end-effector robot with a serial mechanism as human forearm and upper arm to decrease the intervention to patients' movement. The end-effector upper limb rehabilitation robot for assessment (EEULRbot, shown in Figure 1(b)) had two joints as human shoulder and elbow joints with angle sensors and had a two-dimensional force sensor at the end-effector to acquire patients' output force during movement [26].

The rehabilitation robot was designed as three modes: the passive mode, the robot guiding patients by the designed training trajectory; the active mode, the robot guided by patients with no resistances; and the assisted-as-needed mode, the robot helping patients as needed. The interactive environment between human and the robot was equivalent to the spring-damping system, and the control law was described in detail in our previous study [26]. The active mode was designed as a training mode for patients who had a voluntary upper limb movement ability as well as an assessment mode for all patients. The rehabilitation robot as a function assessment equipment was accomplished in the active mode, which was easier than exoskeleton robots.

**Figure 3:**
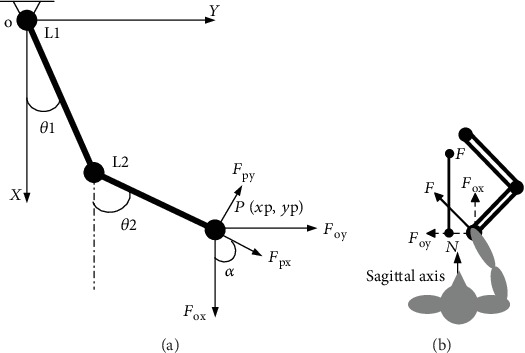
The kinematics analysis of the robot and the direction of interactive force: (a) kinematics analysis and (b) the direction of interactive force.

During the function assessment process, the end of the rehabilitation robot was guided by a patient's hand and the robot recorded the movement characteristics. The rehabilitation robot calculated its shoulder and elbow joints' movement to decrease resistance to the patients' movement according to the inverse dynamic analysis of the robot (shown in Figure 3(a)), described in detail in the previous study [26].

### 2.3. Motor Function Assessment Experiment with the EEULRbot

#### 2.3.1. Subjects

Eight patients with unilateral hemiplegia in the upper limb and nine normal people (years: 26 ± 9, BMI: 21.22 ± 2.04) were recruited during the motor function assessment with EEULRbot. Three of the patients had lesions on the left hemisphere, and the other five had lesions on the right hemisphere. All the patients were evaluated by Fugl-Meyer Assessment scores for upper limb and Activity of Daily Living scores as shown in Table 2.

The criteria for recruitment in this experiment were (1) the first onset of stroke, diagnosed with definite lesions on hemisphere by CT or MRI; (2) capable to resist the upper limb gravity by himself/herself and can move his/her shoulder and elbow actively; (3) without remarkable spasm at elbow joint, with Modified Ashworth Scores ≤ 2 and Brunnstrom Scale≧IV; (4) capable to understand experimenter's request; (5) age between 18 and 80 years; (6) no severe inflammation, pathological injury, and malformation in the paretic arm; (7) no severe visual impairment; and (8) no acute conditions.

All subjects were provided the informed consent form for the experiment, and the experiment was approved by the Medical Ethics Committee of the Affiliated Hospital of National Research Center for Rehabilitation Technical Aids.

#### 2.3.2. Experiment Approach

Participants were required to complete a point-to-point reaching forward and backward movement and a circle drawing movement as shown in Figure 1(a). To make the robot safe for patients, the point-to-point reaching line and circle drawing lines were customized by the patients themselves and their physical therapy. The customized training lines were in the range of patients' movement range, which would not make secondary damage. In case of emergency, it was designed an emergency stop button on the robot, which was easy to reach for the physical therapy but out of range for patients themselves.

The two points were determined by patients' largest range of movement with no trunk movement. The nearest point was decided by the position of the hand when the participant was sitting before a table with his/her shoulder abduction 75°, shoulder flexion 40°, and elbow flexion 90°. The farthest point was decided by the posture that the participant extended his/her elbow to the maximum extension angle and flexed his/her shoulder to the maximum flexion angle along the sagittal axis with no trunk movement. The definition of shoulder abduction and flexion, as well as elbow flexion, was described in Figure 1(c). The shoulder flexion angle was defined as the angle between the projection of the upper limb on the sagittal plane (the xOz coordinate plane) and the coordinate axes (the *x*-axes). The shoulder abduction angle was defined as the angle between the projection of the upper limb on the coronal plane (the xOy coordinate plane) and the coordinate axes (the *x*-axes). The elbow flexion angle was defined as the angle between the forearm and upper limb on the plane determined by the forearm and upper limb.

The task of point-to-point reaching movement was forward and backward line drawing between the nearest point and the farthest point. The task of circle drawing was clockwise or anticlockwise circle drawing from the nearest point to farthest point and then to the nearest point again. The direction of the circle drawing was decided according to patients' lesion location, and healthy participants' circle drawing was a clockwise circle. Clockwise circle drawing was for patients with a lesion at the left hemisphere and anticlockwise circle drawing was for patients with a lesion at the right hemisphere. Every participant was required to perform each task 5 trials and rest 5 seconds between each trial. Each trail should be completed with the patients' physical therapy's accompany, and before the experiments, the physical therapy should help the patients to stretch their arms.

The connection between participants and the robot was the trajectory of the handle and the interactive force at the handle. During the task performance, the information of handle movement and the interactive force was recorded, which was used to describe the participants' movement characteristics. The trajectory of the handle was calculated based on data of the robot shoulder and elbow joint angle sensor (Maxon Encoder, Switzerland Germany,), and the interactive force was measured by the two-dimensional force sensor (Baisen, Shijiazhuang, China). The different data were recorded synchronously at 100 Hz; namely, the frequency of the recorded data during movement was 100.

#### 2.3.3. Data Processing

The trajectory of the handle and the interactive force were recorded. The range, accuracy, and velocity, as well as the acceleration of participants' movement, can be calculated by the trajectory data. The force maximum and force distribution characteristics during movement can be calculated by the force data. The trajectory of the handle can be calculated by the kinematics of the robot according to formula (2). The force data should be transformed from the force sensor relative coordinate system to the movement coordinate system. The transformation of the force can be calculated according to formula (3). The analysis of robot kinematics is shown in Figure 3(a).

*P* represented the handle point, and (*x*_p_, *y*_p_) represented the position coordinates of *P* in the XOY coordinate. *θ*_1_, *θ*_2_ represented the angle of the robot upper limb and forearm to *xX*-axis. *L*_1_, *L*_2_ represented the length of the robot upper limb and forearm. *F*_px_, *F*_py_ represented the measured interactive force in the relative coordinate system of the force sensor. *α* represented the angle of the relative coordinate system and the XOY coordinate system, *α* = *θ*_2_.

(2) $$\begin{array}{c} \left(\begin{array}{c} x_{\text{p}}\\ y_{\text{p}} \end{array}\right)=\left[\begin{array}{cc} \cos\theta_{1} & \cos\theta_{2}\\ \sin\theta_{1} & \sin\theta_{2} \end{array}\right]\left(\begin{array}{c} L_{1}\\ L_{2} \end{array}\right). \end{array}$$

(3) $$\begin{array}{c} \left(\begin{array}{c} F_{\text{ox}}\\ F_{\text{oy}} \end{array}\right)=\left[\begin{array}{cc} -\sin\alpha & \cos\alpha \\ \cos\alpha & \sin\alpha \end{array}\right]\left(\begin{array}{c} F_{px}\\ F_{py} \end{array}\right). \end{array}$$

**Figure 4:**
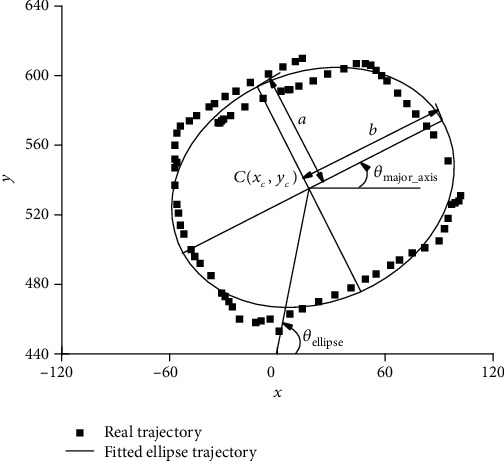
The fitted ellipse trajectory during circle drawing.

*(1) The Range of Movement*. The range of movement reflected participants' shoulder and elbow movement range. The ellipse was fitted based on the trajectory of the handle during circle drawing, shown in Figure 4. The characteristic parameters of the fitting ellipse can describe the movement range. The elliptic equation was shown in formula (4), and its parameters' meaning and calculation method were described in Figure 4 and Table 3.

(4) $$\begin{array}{c} Ax^{2}+Bxy+Cy^{2}+Dx+Ey+1=0. \end{array}$$

Formula (4) was the general equations of the fitted ellipse.

(5) $$\begin{array}{c} x_{\text{c}}=\frac{BE-2CD}{4AC-B^{2}},\\ y_{\text{c}}=\frac{BD-2AE}{4AC-B^{2}}. \end{array}$$

*C*(x_c_, *y*_c_) represented the center of fitted ellipse.

(6) $$\begin{array}{c} r=A{x_{\text{c}}}^{2}+Bx_{\text{c}}y_{\text{c}}+C{y_{\text{c}}}^{2}-1. \end{array}$$

*r* represented a transition calculated parameter to make the expression of parameters in Table 3 simple and clear.

*S*_real_ represented the area of the real trajectory during the circle drawing movement, and *S*_task_ represented the area of the target trajectory, which was the circle decided by the farthest and nearest point. The area ratio of real and target circle drawing *S*_ratio_ described the capacity to perform the entire movement of the maximum movement range. *R*_ratio_, the ratio of the fitted ellipse' two semi-axis described the roundness of the real trajectory. *θ*_major_axis_, the inclination angle of the long semi-axis, and *θ*_ellipse_, the rotation angle of the fitted ellipse center, described the patients' preferred movement direction during the circle drawing.

*(2) The Accuracy of Movement*. The distance between the target movement trajectory and the real performed trajectory can be used to describe the movement accuracy. During the point-to-point reaching movement, the distance was calculated by the distance from the real point to the target line. During the circle drawing movement, the distance was defined as the distance from the real point to the nearest circle point along the direction of the diameter. The average distance during the entire performance was used to describe the movement accuracy. Besides, the average distance during circle drawing movement was normalized by dividing the circle radius.

All the accuracy parameters were calculated according to formula (7)-formula (12).

(7) $$\begin{array}{c} {\text{disp}}_{\text{l}}=\frac{Ax_{\text{p}}+By_{\text{p}}+C}{\sqrt{A^{2}+B^{2}}}. \end{array}$$

(*x*_p_, *y*_p_) represented the handle point coordinates. disp_l_ represented the distance during point-to-point reaching movement; *A*, *B*, and *C* represented the parameters in the reaching line equation *Ax* + *By* + *C* = 0 in the XOY coordinate system shown in Figure 4.

(8) $$\begin{array}{c} {\text{disp}}_{\text{c}}=\sqrt{{\left(x_{\text{p}}-x_{\text{c}}\right)}^{2}+{\left(y_{\text{p}}-y_{\text{c}}\right)}^{2}}-r. \end{array}$$

(*x*_c_, *y*_c_) represented the circle center point coordinates in XOY coordinate system. *r* represented the target circle radius. *disp*_c_ represented the distance during the circle drawing movement.

(9) $$\begin{array}{c} {\text{disp}}_{\text{average}}=\frac{\sum_{i=1}^{n}disp\left(i\right)}{n}. \end{array}$$

disp_average_ represented the average distance during the movement. *n* represented the number of the recorded point data. disp meant disp_l_ or disp_c_.

To meticulously analyze the reaching movement, the point-to-point reaching movement was divided into forward movement (FWM) and backward movement (BWM). To make it possible to compare the movement characteristics among different participants, the distance during circle drawing movement was normalized by the target circle radius *r*, according to

(10) $$\begin{array}{c} R_{\text{dispc}}=\frac{\left|{\text{disp}}_{\text{c}}\right|}{r}\;\left(\text{if }\left|{\text{disp}}_{\text{c}}\right|\geq r,\left|{\text{disp}}_{\text{c}}\right|=r\right),\\ {\bar{R}}_{\text{dispc}}=\frac{\sum_{i=1}^{n}R_{\text{dispc}}\left(i\right)}{n}. \end{array}$$

*R*_dispc_ represented the normalized distance during the circle drawing movement. If ∣disp_c_∣ ≥ *r*, ∣disp_c_∣ = *r*, then 0 ≤ *R*_dispc_ ≤ 1. ${\bar{R}}_{\text{dispc}}$ represented the average normalized distance during the circle drawing. *n* represented the number of recorded point data.

To directly describe the movement accuracy, the derived parameter ${\bar{R}}_{\text{accuracyc}}$ was calculated according to formula (11) and formula (12).$\;{\bar{R}}_{\text{accuracy}}$ represented the average movement accuracy during the circle drawing. *n* represented the number of recorded point data.

(11) $$\begin{array}{c} R_{\text{accuracyc}}=1-R_{\text{dispc}}, \end{array}$$

(12) $$\begin{array}{c} {\bar{R}}_{\text{accuracyc}}=\frac{\sum_{i=1}^{n}R_{\text{accuracyc}}\left(i\right)}{n}. \end{array}$$

*(3) The Smoothness of Movement*. The number of peaks in movement velocity and acceleration can describe the smoothness of movement.

The velocity and acceleration of movement were calculated according to formulas (13) and (16):

(13) $$\begin{array}{c} v_{i}=\frac{\sqrt{{\left(x_{i+1}-x_{i}\right)}^{2}+{\left(y_{i+1}-y_{i}\right)}^{2}}}{t_{i+1}-t_{i}},\;\left(i=1,2,3,\cdots ,n-1\right). \end{array}$$

*v*_*i*_ represented the velocity at *t*_*i*_, (*x*_*i*+1_, *y*_*i*+1_) represented the hand point position coordinate at *t*_*i*+1_, and (*x*_*i*_, *y*_*i*_) represented the point coordinate at *t*_*i*_. *n* represented the number of recorded point data.

(14) $$\begin{array}{c} a_{i}=\frac{v_{i+1}-v_{i}}{t_{i+1}-t_{i}},\;\left(i=1,2,3,\cdots ,n-1\right). \end{array}$$

*v*_*i*+1_ represented the velocity at *t*_*i*+1_, and *a*_*i*_ represented the handle acceleration at *t*_*i*_. *n* represented the number of recorded point data.

The average and max velocities were calculated to describe the average and max movement ability, according to

(15) $$\begin{array}{c} v_{\max}=\max\left(v_{i}\right),\;\left(i=1,2,3,\cdots ,n\right), \end{array}$$

(16) $$\begin{array}{c} v_{\text{average}}=\frac{\sum_{i=1}^{n}v_{i}}{n}. \end{array}$$

*v*_max_ represented the maximum velocity during the movement, and *v*_average_ represented the average velocity during the movement. *n* represented the number of recorded point data.

The number of velocity peaks in movement velocity was recorded according to the velocity curves during movement.

*(4) The Maximum, Range of the Interactive Force, and the Ratio of the Force along the Sagittal Axis*. The interactive force demonstrated the participants' output force capability. The maximum and range of the interactive force described participants' capacity of performing force, which was the output of their muscles. The ratio of the force along the sagittal axis was analyzed during point-to-point drawing to describe participants' ability to control the output force along the direction of the sagittal axis, which was shown in Figure 3(b).

All the parameters of interactive force were calculated according to formula (17)-formula (21):

(17) $$\begin{array}{c} R_{\text{Fx}}\left(i\right)=\frac{F_{\text{ox}}\left(i\right)}{F_{oy}\left(i\right)},\;\left(i=1,2,3,\cdots ,n\right), \end{array}$$

(18) $$\begin{array}{c} {\bar{R}}_{\text{Fx}}=\frac{\sum_{i=1}^{n}R_{\text{Fx}}\left(i\right)}{n}. \end{array}$$

*F*_ox_ and *F*_oy_ represented the interactive force in Figure 3(b); *F*_ox_ was along the sagittal axis, which was the direction of the point-to-point target line. ${\bar{R}}_{\text{Fx}}$ represented the ratio of *F*_ox_, the effective force of participants during the point-to-point reaching movement. *n* represented the number of recorded point data.

The maximum and range of force were calculated to describe the max output force ability of patients during movement, according to

(19) $$\begin{array}{c} \text{F}\left(\text{i}\right)=\sqrt{F_{\text{ox}}{\left(i\right)}^{2}+F_{\text{oy}}{\left(i\right)}^{2}},\;\left(i=1,2,3,\cdots ,n\right), \end{array}$$

(20) $$\begin{array}{c} F_{\max}=\max\left(F\left(i\right)\right),\;\left(i=1,2,3,\cdots ,n\right), \end{array}$$

(21) $$\begin{array}{c} F_{\text{range}}=\max\left(F\left(i\right)\right)-\min\left(F\right)\;\;\left(i=1,2,3,\cdots ,n\right). \end{array}$$

*n* represented the number of recorded point data.

*(5) Time Normalization*. The trajectory of the handle and the interactive force were normalized to 100 points in the time duration of the movement in order to analyze the changes between participants during the entire movement. It was first normalized by time according to

(22) $$\begin{array}{c} A_{i}=A\left(\frac{t_{\text{i}}}{t_{0}}\right),\;\left(i=1,2,3,\cdots \text{n}\right). \end{array}$$

*t*_0_ was the time duration of the entire movement, and *t*_*i*_ was the time of the point i recorded by the robot in the movement. *A*(*t*_i_/*t*_0_) represented the previous calculated dynamic parameters: distance, velocity, acceleration, interactive force, and the ratio of the force along the sagittal axis. *n* represented the number of recorded point data. Then, *A*_*i*_ was interpolated to 100 points.

*(6) Quantitative Relationship between Parameters and Motor Function Assessment Scores*. To establish a quantitative assessment method for patients' motor function, firstly, the parameters between stroke patients and healthy subjects were compared to find the parameters that could unique patients from healthy subjects. Then, a multivariate fitting method was used to develop the relationship between unique parameters and patients' motor function assessment scores.

*(7) Statistical Analysis*. All the statistical analysis was calculated in IBM SPSS STATISTICS 22. The difference level of parameters between stroke patients and healthy subjects were analyzed by Welch's *t*-test, which was more reliable when the two samples had unequal sample sizes (the sample sizes of the two groups in this paper were 8 and 9) [27]. The significance level was set at 0.05 (*p* < 0.05). If the parameter was different significantly in statistics between stroke patients and healthy subjects, the effect size of the difference was evaluated by Cohen's *d* [28].

## 3. Results

### 3.1. Characteristics of Patients' Movement

#### 3.1.1. The Range of the Movement

The range of the movement was demonstrated by *R*_ratio_, *θ*_major_axis_, *θ*_ellipse_, *S*_ratio_, and their derived parameters |*R*_ratio_ − 1| and |*S*_ratio_ − 1| during circle drawing movement, which were shown in Table 4.

#### 3.1.2. The Accuracy of Movement

**Figure 5:**
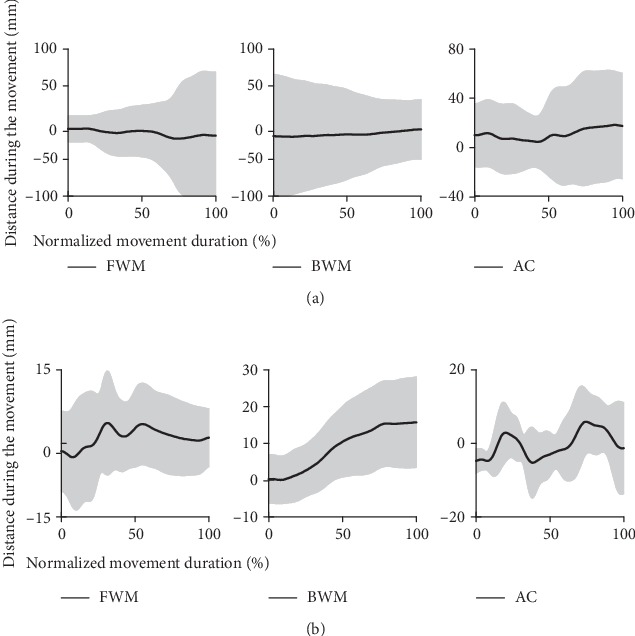
The distance during movement of stroke patients and healthy subjects: (a) the distance during movement of stroke patients and (b) the distance during movement of healthy subjects.

The accuracy of movement was described by the movement average distance during forward point-to-point disp_average_FWM_, backward point-to-point disp_average_BWM_, and circle drawing movement disp_average_AC_ and ${\bar{R}}_{\text{dispc}}$. The distance during movement was shown in Figure 5. FWM represented the forward point-to-point reaching movement, BWM represented the backward point-to-point reaching movement, and AC represented the active circle drawing movement. The black solid line was the average value among the subjects, and the gray-shaded area was one positive and negative standard deviation.

disp_average_FWM_, disp_average_BWM_, and disp_average_AC_ were the absolute value of distance. ${\bar{R}}_{\text{dispc}}$ was the relative value of the distance to the circle radius. ${\bar{R}}_{\text{accuracyc}}$ was the derived parameter which directly reflected the movement accuracy. The bigger the distance value was, the poorer the movement accuracy was. The distance in stroke patients was much bigger than that in healthy subjects. The stroke patients performed the characteristics that the distance decreased during the forward point-to-point reaching movement and increased during the backward point-to-point reaching movement. The distance in stroke patients during both the point-to-point reaching movement and the circle drawing movement (disp_average_FWM_, 26.88 mm; disp_average_BWM_, 25.60 mm; disp_average_AC_, 22.42 mm) was nearly one to three times more than that in healthy subjects (disp_average_FWM_, 6.88 mm; disp_average_BWM_, 10.63 mm; disp_average_AC_, 7.36 mm) (shown in Table 5). Besides, the standard deviation of distance in stroke patients was much bigger than that in healthy subjects. The movement accuracy parameter ${\bar{R}}_{\text{accuracyc}}$ in stroke patients (${\bar{R}}_{\text{accuracyc}}$, 0.49) was significantly smaller than that in healthy subjects (${\bar{R}}_{\text{accuracyc}}$, 0.91) (*F* = 36.253, *p* ≤ 0.001) and it had a medium effect size with Cohen's *d* (*d* = 0.732).

#### 3.1.3. The Smoothness of Movement

**Figure 6:**
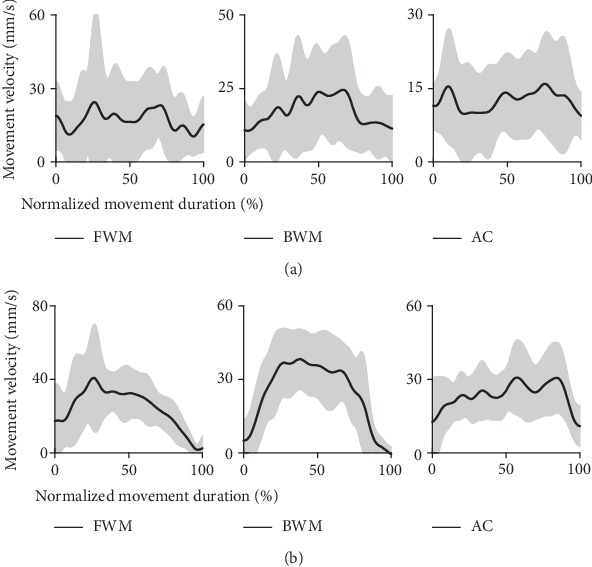
The movement velocity during movement: (a) the movement velocity during movement of stroke patients and (b) the movement velocity during movement of healthy subjects.

Movement smoothness was described by the changes in movement velocity and acceleration. The movement velocity during movement is shown in Figure 6, and the maximum velocity, the mean velocity, and the number of peaks, and valleys of velocity are illustrated in Table 6.

**Figure 7:**
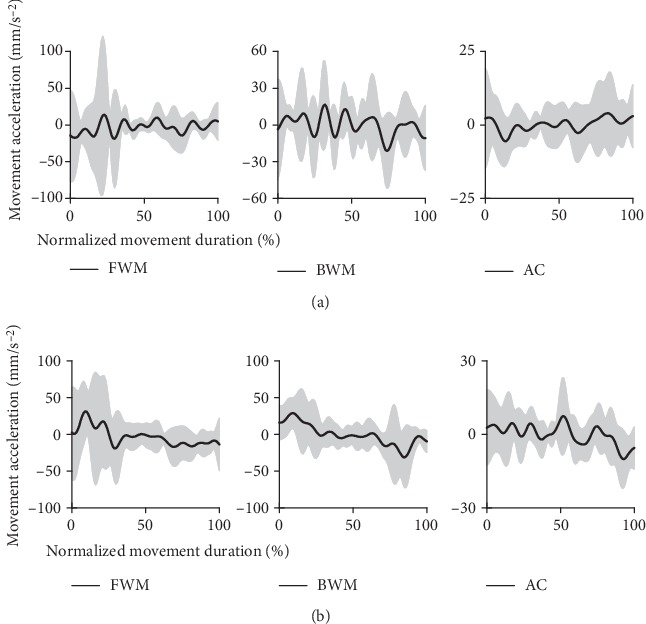
The movement acceleration during movement: (a) the acceleration during movement of stroke patients and (b) the acceleration during movement of healthy subjects.

The changes during movement were also described by the acceleration. Movement acceleration was shown in Figure 7. There were more changes in the movement acceleration of stroke patients than that of healthy subjects. The more changes in movement acceleration demonstrated that the movement smoothness was poorer in stroke patients.

#### 3.1.4. Maximum, Range of the Interactive Force, and the Ratio of Force along the Sagittal Axis

*F*_max_ was the maximum value of the interactive force during the movement, *F*_range_ was the range of the interactive force during the movement, and ${\bar{R}}_{\text{Fx}}$ was the ratio of the interactive force along the sagittal axis. The interactive force parameters were shown in Table 7.

*F*_range_ in stroke patients during the forward point-to-point movement was significantly bigger with a big effect size than that in healthy subjects (*F* = 16.365, *p* = 0.001, *d* = 0.522 (large)). *F*_range_ in stroke patients during the backward point-to-point movement was slight smaller than that in healthy subjects. *F*_max_ in stroke patients during the forward point-to-point movement and the circle drawing movement were bigger than those in healthy subjects. *F*_max_ in stroke patients during the backward point-to-point movement was smaller than that in healthy subjects. ${\bar{R}}_{\text{Fx}}$ in stroke patients during movement was smaller than that in healthy subjects, especially ${\bar{R}}_{\text{Fx}}$ in stroke patients during the backward point-to-point movement was significantly smaller with a very large effect size than that in healthy subjects (FWM, *F* = 43.155, *p* ≤ 0.001, *d* = 0.742 (large); BWM, *F* = 126.596, *p* ≤ 0.001, *d* = 0.894 (very large)).

### 3.2. Quantitative Relationship between Parameters and Motor Function Assessment Scores

By analyzing the parameters, it was concluded that |*R*_ratio_ − 1|, |*S*_ratio_ − 1|, and ${\bar{R}}_{\text{accuracyc}}$ in circle drawing movement and ${\bar{R}}_{\text{Fx}}$ in the backward point-to-point movement were dimensionless and unique parameters that were different remarkably between stroke patients and healthy subjects. During the experiment, patients were assessed with Fugl-Meyer scales (FM) for the upper limb which was the commonly used method for assessment of upper limb motor function. First, the multivariate fitting function with the previous four parameters and FM was analyzed. |*R*_ratio_ − 1| was excluded for its high collinearity with FM (VIF = 15.24 > 10, shown in Table 8).

It was analyzed that the multivariate fitting function of the relationship between the other three parameters |*S*_ratio_ − 1|, ${\bar{R}}_{\text{accuracyc}}$ in circle drawing movement, ${\bar{R}}_{\text{Fx}}$ in backward point-to-point movement, and FM scores. The collinearity of the three assessment indicators was shown in Table 9. The VIF of all the indicators was bigger than 1 and smaller than 10, which represented there were no collinearity symptoms. The fitting function can be described as formula (9).

(23) $$\begin{array}{c} \text{y}\left(\text{FM}\right)=-14.22-4.64\times \left|S_{\text{ratio}}-1\right|+4.32\times {\bar{R}}_{\text{accuracyc}}+155.30\times {\bar{R}}_{\text{Fx}}\left(\text{R}2=0.91,p=0.015\right). \end{array}$$

## 4. Discussion

### 4.1. Characteristics of Patients' Movement

Stroke patients had limited control ability during the movement with much changes in the direction for their decreased analysis ability of the environment and their decreased control ability of coordinate movement during multiple joints.

#### 4.1.1. The Range of the Movement

Because of bicep muscle's high tension, patients with a stroke had a symptom of elbow flexion, which made it difficult to extend their elbow [29–31]. During the circle drawing movement, patients made the trajectory out of the target during elbow flexion and inner of the target during elbow extension, which may increase the deviation of the real trajectory to the target, demonstrated by |*S*_ratio_ − 1| in this study. *S*_ratio_ in stroke patients was deviated more from 1.0, which meant the real movement area was a bigger deviation than the target movement area. This may be caused by patients' movement out or inner of the target trajectory, especially in the preferred movement direction closer to the coronal axis and the difficult movement direction along the sagittal axis.

#### 4.1.2. The Accuracy and Smoothness of the Movement

Due to the poor control of the coordinated movement of multiple joints, stroke patients performed poorer movement accuracy and movement smoothness, which are demonstrated by the characteristics that the distance of the real trajectory and the target trajectory in stroke patients was bigger and that there were more velocity peaks and valleys in stroke patients' movement.

Patients' poorer control ability of elbow extension and their decreased movement control ability during the distal movement [32] may result in a bigger distance during the forward point-to-point reaching movement. Besides, patients with a stroke usually had abnormal joint movement patterns between shoulder and elbow, namely the movement of shoulder abduction came along with elbow flexion and shoulder adduction came along with elbow extension [33–35], which may also increase the distance during target line and circle drawing movement.

There were more submovement patterns during continuous arm motion for stroke patients because of the residual function of the damaged cortex or subcortex [36]. The submovements occurred near the maximum capacity of the neuromuscular system during the movements when patients made efforts to increase the movement velocity, which may result in the more velocity peaks and valleys. Fewer peaks in speed represented fewer periods of acceleration and deceleration, which would make a smoother movement [17]. In our study, the number of velocity peaks and valleys was more than that in healthy subjects, which was consistent with Colombo et al. [15] and Panerese et al. [16].

#### 4.1.3. Maximum, Range of the Interactive Force, and the Ratio of Force along the Sagittal Axis

Despite the decreased control ability of movement accuracy and smoothness, patients had decreased control ability of force value and force direction, demonstrated by a bigger *F*_range_ and *F*_max_ during the forward point-to-point reaching movement. Because of the decreased control ability of elbow extension during the distal movement, patients may increase the elbow extension moment to overcome the elbow flexion stiffness and they cannot timely decrease the extension moment at target points, leading to a bigger *F*_range_ and *F*_max_.

Besides, stroke patients performed less force along the sagittal axis (${\bar{R}}_{\text{Fx}}$) than healthy subjects, during the point-to-point movement. This may be caused by the poor coordinate movement of shoulder and elbow joints. Patients usually recovered the control ability of elbow more difficult than the shoulder joint [37], and there were abnormal movement patterns between shoulder and elbow joints [35]. The ratio of force along the sagittal axis was the output of coordinated shoulder and elbow movement. Once the shoulder abduction movement or the elbow flexion was bigger than the required, it may result in a big force at the handle along the lateral force during line movements, leading to a lower ratio force along the sagittal axis.

### 4.2. Quantitative Relationship between Parameters and Motor Function Assessment Scores

In clinics, motor function assessment of stroke patients included the movement range and movement accuracy such as touching one's nose by the hemiplegic hand. So during the quantitative assessment, it should include the unique parameters in movement range, accuracy, as well as unique force characteristics.

By analyzing the multivariable regression of the relationship between the unique parameters, |*S*_ratio_ − 1|, ${\bar{R}}_{\text{accuracyc}}$ in the circle drawing movement and ${\bar{R}}_{\text{Fx}}$ in the backward point-to-point movement, and FM scores, we got the fitting function for the quantitative assessment method. From the fitting function, it can be concluded that FM was negatively correlated with |*S*_ratio_ − 1| and positively correlated with ${\bar{R}}_{\text{accuracyc}}$ and ${\bar{R}}_{\text{Fx}}$. The fitting function was in a good fitting with *R*^2^ = 0.91. Based on the quantitative parameters, the fitting function can be used to calculate the FM scores as a quantitative assessment method for upper limb motor function.

## 5. Limitation

This study had made researches on the unique parameters which can be used as the assessment parameters to describe the motor function. But the number of the research was a little small, which may influence the validation of the method. It should involve more patients and make a repeated measurement of the same patients to verify reproducibility and validation in the future.

## 6. Conclusions

The basic movement in our daily life was considered as point-to-point reaching movement. To assess the coordinate movement of stroke patients, the principal movement patterns of function assessment included the circle drawing movement. The circle drawing movement was verified to involve more muscle co-contraction between shoulder flexion/extension, shoulder abduction/adduction, and elbow flexion/extension movement in this paper. According to the principal movement patterns, this paper proposed an end-effector rehabilitation robot with a serial mechanism. This rehabilitation robot can be used to establish the function assessment process without other complicated equipment.

It involved eight stroke patients to perform the principal movement patterns with the robot recorded the trajectory and the interactive force during the movement in the project. The characteristics of the movement, such as the range of movement, the accuracy of the movement, the smoothness of the movement, and the interactive force parameters, were calculated and analyzed in this paper. It can be concluded that |*S*_ratio_ − 1|, ${\bar{R}}_{\text{accuracyc}}$ in the circle drawing movement and ${\bar{R}}_{\text{Fx}}$ in the backward point-to-point movement were different remarkably between patients and healthy subjects.

Besides, this paper analyzed the relationship between the unique movement parameters and the Fugl-Meyer scores and concluded that |*S*_ratio_ − 1|, ${\bar{R}}_{\text{accuracyc}}$ in the circle drawing movement, and ${\bar{R}}_{\text{Fx}}$ in the backward point-to-point movement had a remarkable relationship with the Fugl-Meyer scores. The fitting function between the parameters and the Fugl-Meyer scores for upper limb can be used as the quantitative assessment method, which would make the assessment process timely by combining the assessment process with the rehabilitation training process and further the application of rehabilitation robots in clinics.

## Figures and Tables

**Table 1:** Primary agonist and antagonist for shoulder and elbow joints.

Movement	Primary agonist and antagonist
Shoulder flexion/extension	Trapezius (TR)-Latissimus dorsi (LA)
Shoulder abduction/adduction	Deltoid muscles (DE)-Latissimus dorsi (LA)
Shoulder internal/external rotation	Infraspinatus (IN)-Subscapularis(SUP)
Elbow flexion/extension	Biceps brachii(BB)-triceps brachii(TB)

**Table 2:** The clinical information of patients with stroke.

Number	Years	Gender	Lesion location (left or right hemisphere)	Days since stroke	FM for upper
S1	64	Male	Right	180	11
S2	35	Male	Right	62	12
S3	21	Male	Left	107	17
S4	54	Male	Left	128	19
S5	32	Male	Right	41	43
S6	38	Male	Right	180	45
S7	43	Male	Left	54	53
S8	37	Male	Right	46	59

FM scores for upper, Fugl-Meyer assessment scores for the upper limb. The total scores in FM for the upper limb was 66.

**Table 3:** Parameters of the fitted ellipse trajectory.

Parameters	Calculation
Long semi-axis	$\text{a}=\sqrt{\frac{r}{A}}$
Short semi-axis	$\text{b}=\sqrt{\frac{4Ar}{\left(4AC-B^{2}\right)}}$
The ratio of two semi-axis	$R_{\text{ratio}}=\frac{b}{a}$
The inclination angle of long semi-axis	$\theta_{\text{major}\_\text{axis}}=\frac{1}{2}\tan^{-1}\frac{B}{A-C}$
The rotation angle of the ellipse center	$\theta_{\text{ellipse}}=\tan^{-1}\frac{y_{c}}{x_{c}}$
The area ratio of the real and the target circle drawing	$S_{\text{ratio}}=\frac{S_{\text{real}}}{S_{\text{task}}}$

**Table 4:** Parameters of the range of the movement during circle drawing movement.

Parameters	Healthy subjects (*M* ± STD.)	Stroke patients (*M* ± STD.)
*R*_ratio_	0.94 ± 0.03	0.92 ± 0.19^∗^
|*R*_ratio_ − 1|	0.06 ± 0.03	0.14±0.15^∗∗^
*S*_ratio_	0.99 ± 0.08	1.20 ± 0.50^∗^
|*S*_ratio_ − 1|	0.07 ± 0.04	0.32±0.42^∗∗^
*θ*_major_axis_ (°)	90.42 ± 1.45	92.39 ± 5.61
*θ*_ellipse_ (°)	69.71 ± 6.84	55.73 ± 22.20

M: mean value; STD: standard deviation. *R*_ratio_ was the ratio of the short semi-axis and long semi-axis of the fitted ellipse of real trajectory, which represented the degree of the ellipse close to a circle. If *R*_ratio_ was closer to 1, the real trajectory would be closer to a circle. *R*_ratio_ in stroke patients was a little smaller than that in healthy subjects (*F* = 0.103, *p* = 0.037). |*R*_ratio_ − 1| between stroke patients and healthy subjects was different from each other (*F* = 2.516, *p* = 0.007), and |*R*_ratio_ − 1| in stroke patients was larger than that in healthy subjects, which represented that patients' movement trajectory deviated more from a circle than healthy subjects. *S*_ratio_ was the ratio of the real trajectory area and the target circle area, which represented the ratio of the real movement range to the ideal movement range. If *S*_ratio_ was closer to 1, the real movement range would be closer to the ideal range. *S*_ratio_ in stroke patients was larger than that in healthy subjects (*F* = 1.523, *p* = 0.039), and |*S*_ratio_ − 1| in stroke patients was much larger than that in healthy subjects (*F* = 3.089, *p* = 0.001). *θ*_major_axis_ and *θ*_ellipse_ represented, respectively, the inclination angle of the long semi-axis and the rotation angle of the fitted ellipse' center, which described the participants' preferred movement direction. *θ*_major_axis_ (92.39°) in stroke patients was a little larger than that in healthy subjects (90.42°) (*F* = 0.930, *p* = 0.364). *θ*_ellipse_ in stroke patients (55.73°) was much smaller than that in healthy subjects (69.71°) (*F* = 2.925, *p* = 0.125). Both of *θ*_major_axis_ and *θ*_ellipse_ demonstrated that patients preferred to move in a direction that closer to the coronal axis which was parallel to the *x* coordinate axis shown in Figure 4.

**Table 5:** Parameters of movement accuracy during movement.

Parameters	Healthy subjects (*M* ± STD.)	Stroke patients (*M* ± STD.)
disp_average_FWM_ (mm)	6.88 ± 3.96	26.88 ± 30.19
disp_average_BWM_ (mm)	10.63 ± 7.04	25.60 ± 34.05
disp_average_AC_ (mm)	7.36 ± 1.85	22.42±5.93^∗∗^
${\bar{R}}_{\text{dispc}}$	0.09 ± 0.03	0.52±0.21^∗∗^
${\bar{R}}_{\text{accuracyc}}$	0.91 ± 0.03	0.49±0.19^∗∗^

^∗∗^ represented *p* < 0.005.

**Table 6:** The average velocity and max velocity during movement.

	*v*_average_ (mm/s) (*M*(STD))		*v*_max_ (mm/s) (*M*(STD))		Number of peaks and valleys (*M*(STD))	
	Healthy subjects	Stroke patients	Healthy subjects	Stroke patients	Healthy subjects	Stroke patients
FWM	24.24 (7.71)	17.06 (9.30)	92.28 (64.54)	108.59 (47.43)	7 (2)	8^∗∗^ (2)
BWM	24.04 (9.66)	18.00 (9.82)	63.25 (20.70)	76.3 (25.76)	6 (2)	8^∗^ (2)
AC	23.57 (8.62)	12.58^∗^ (7.76)	96.19 (53.91)	112.38 (64.41)	6 (1)	8^∗∗^ (1)

∗∗ represented *p* < 0.005; ∗ represented *p* < 0.05. FWM represented the forward point-to-point reaching movement, BWM represented the backward point-to-point reaching movement, and AC represented the active circle drawing movement. Healthy subjects performed that velocity increased at the early movement, then increased to a stabilized value during the intermediate stage of the movement, and decreased at the last movement shown in Figure 6(b). Patients also performed similar velocity changes during the backward point-to-point movement but not performed the velocity changes during the forward point-to-point movement and circle drawing movement described in Figure 6(a). The average movement velocity during the entire movement in stroke patients was smaller than that in healthy subjects, especially the average movement velocity in stroke patients (12.58) was smaller significantly in statistics with a small effect size (*F* = 7.552, *p* = 0.015, *d* = 0.335) than that in healthy subjects (23.57) during circle drawing movement. The max velocity in stroke patients was bigger than that in healthy subjects. The number of peaks and valleys during movement in stroke patients (8) was bigger than that in healthy subjects (6) with a significant difference in statistics (FWM, *F* = 15.478, *p* = 0.001, *d* = 0.508 (median); BWM, *F* = 7.333, *p* = 0.016, *d* = 0.328 (small); AC, *F* = 22.766, *p* ≤ 0.001, *d* = 0.603 (large)). Stroke patients performed more velocity changes than healthy subjects during movement.

**Table 7:** The interactive force parameters during movement.

	*F*_range_ (*N*) (*M* ± STD)		*F*_max_ (*N*) (*M* ± STD)		${\bar{R}}_{\text{Fx}}$ (*M* ± STD)	
	Healthy subjects	Stroke patients	Healthy subjects	Stroke patients	Healthy subjects	Stroke patients
FWM	10.09 ± 4.43	19.58±5.24^∗∗^	14.74 ± 5.58	22.76 ± 5.74^∗^	0.73 ± 0.11	0.36±0.13^∗∗^
BWM	17.66 ± 21.37	16.72 ± 5.54	23.10 ± 22.18	19.71 ± 5.67	0.76 ± 0.06	0.29±0.11^∗∗^
AC	15.84 ± 9.95	18.74 ± 2.69	18.88 ± 10.67	21.46 ± 2.60	0.49 ± 0.06	0.45 ± 0.09

^∗∗^ *p* < 0.005; ^∗^*p* < 0.05, which demonstrated that the difference between the parameters in stroke patients and that in healthy subjects was significant in statistics.

**Table 8:** The multivariate fitting function of indicators with collinearity test.

Indicators	Coefficient	Collinearity (VIF)
Constant	-42.63	
|*R*_ratio_ − 1|	117.19	15.24
|*S*_ratio_ − 1|	-29.14	7.14
${\bar{R}}_{\text{accuracyc}}$	36.02	9.35
${\bar{R}}_{\text{Fx}}$	172.37	8.70

**Table 9:** The multivariate fitting function of indicators except |*R*_ratio_ − 1| with collinearity test.

Indicators	Coefficient	Collinearity (VIF)
Constant	-14.22	
|*S*_ratio_ − 1|	-4.64	2.08
${\bar{R}}_{\text{accuracyc}}$	4.32	7.09
${\bar{R}}_{\text{Fx}}$	155.30	8.55

## Data Availability

The data is available by the corresponding email or the first author email.

## Supplementary Materials

Supplementary Materials The supplementary materials are the detailed results of the collinearity test in our indicators analysis.
