# Supplementary material for: Quantitative Assessment of Motor Function for Patients with a Stroke by an End-Effector Upper Limb Rehabilitation Robot
Source: Biomed Res Int. 2020 Apr 8;2020:5425741. doi: 10.1155/2020/5425741 (PMC7224306; doi:10.1155/2020/5425741)
Supplement: Supplementary Materials — The supplementary materials are the detailed results of the collinearity test in our indicators analysis. [file 5425741.f1.htm]

xml version="1.0" encoding="UTF-8"?


patients parameters assessment


IBM SPSS Web Report - Output1

(Controls disabled by the system)

- Print
- Open in Simplified View
- Open in Interactive View

- Log

  - Log
- Regression

  - Active Dataset
  - Variables Entered/Removed
  - Model Summary
  - ANOVA
  - Coefficients
  - Collinearity Diagnostics
- Log

  - Log
- Regression

  - Variables Entered/Removed
  - Model Summary
  - ANOVA
  - Coefficients
  - Collinearity Diagnostics

System Default
Classic
Classic Alternate
Cobalt Alternate
Gray
Gray Alternate


A

|  |  |  |  |  |  |
| --- | --- | --- | --- | --- | --- |
|  | |  | |  | |
|  |  |  |  |  |  |
|  |  |  |  |  |  |
|  |  |  |  |  |  |
|  |  |  |  |  |  |
|  |  |  |  |  |  |

Log

GET  
  FILE='F:\博士后工作\论文投稿\康复相关\data\patients parameter assessment.sav'.  
DATASET NAME DataSet1 WINDOW=FRONT.  
REGRESSION  
  /MISSING LISTWISE  
  /STATISTICS COEFF OUTS R ANOVA COLLIN TOL  
  /CRITERIA=PIN(.05) POUT(.10)  
  /NOORIGIN  
  /DEPENDENT FM  
  /METHOD=ENTER rratio1 sratio1 raccuracy fratiobal.

Regression

[DataSet1] F:\博士后工作\论文投稿\康复相关\data\patients parameter assessment.sav

Regression

Variables Entered/RemovedaVariables Entered/Removed, 表, 列标题的 1 级和行标题的 1 级, 具有 4 列和 5 行的表

|  |  |  |  |
| --- | --- | --- | --- |
| Model | Variables Entered | Variables Removed | Method |
| 1 | fratiobal, sratio1, raccuracy, rratio1b | . | Enter |
|  |  |  |  |
| --- | --- | --- | --- |
| a. Dependent Variable: FM | | | |
| b. All requested variables entered. | | | |
|  |  |  |  |

Regression

Model SummaryModel Summary, 表, 列标题的 1 级和行标题的 1 级, 具有 5 列和 4 行的表

|  |  |  |  |  |
| --- | --- | --- | --- | --- |
| Model | R | R Square | Adjusted R Square | Std. Error of the Estimate |
| 1 | .981a | .963 | .913 | 5.782 |
|  |  |  |  |  |
| --- | --- | --- | --- | --- |
| a. Predictors: (Constant), fratiobal, sratio1, raccuracy, rratio1 | | | | |
|  |  |  |  |  |

Regression

ANOVAaANOVA, 表, 列标题的 1 级和行标题的 2 级, 具有 7 列和 7 行的表

|  |  |  |  |  |  |  |
| --- | --- | --- | --- | --- | --- | --- |
| Model | | Sum of Squares | df | Mean Square | F | Sig. |
| 1 | Regression | 2593.577 | 4 | 648.394 | 19.394 | .018b |
|  | Residual | 100.298 | 3 | 33.433 |  |  |
|  | Total | 2693.875 | 7 |  |  |  |
|  |  |  |  |  |  |  |
| --- | --- | --- | --- | --- | --- | --- |
| a. Dependent Variable: FM | | | | | | |
| b. Predictors: (Constant), fratiobal, sratio1, raccuracy, rratio1 | | | | | | |
|  |  |  |  |  |  |  |

Regression

CoefficientsaCoefficients, 表, 列标题的 2 级和行标题的 2 级, 具有 9 列和 9 行的表

|  |  |  |  |  |  |  |  |  |
| --- | --- | --- | --- | --- | --- | --- | --- | --- |
| Model | | Unstandardized Coefficients | | Standardized Coefficients | t | Sig. | Collinearity Statistics | |
|  |  | B | Std. Error | Beta |  |  | Tolerance | VIF |
| 1 | (Constant) | -42.636 | 17.678 |  | -2.412 | .095 |  |  |
|  | rratio1 | 117.191 | 56.244 | .906 | 2.084 | .129 | .066 | 15.243 |
|  | sratio1 | -29.145 | 13.965 | -.621 | -2.087 | .128 | .140 | 7.144 |
|  | raccuracy | 36.029 | 30.921 | .397 | 1.165 | .328 | .107 | 9.352 |
|  | fratiobal | 172.379 | 61.083 | .927 | 2.822 | .067 | .115 | 8.704 |
|  |  |  |  |  |  |  |  |  |
| --- | --- | --- | --- | --- | --- | --- | --- | --- |
| a. Dependent Variable: FM | | | | | | | | |
|  |  |  |  |  |  |  |  |  |

Regression

Collinearity DiagnosticsaCollinearity Diagnostics, 表, 列标题的 2 级和行标题的 2 级, 具有 9 列和 9 行的表

|  |  |  |  |  |  |  |  |  |
| --- | --- | --- | --- | --- | --- | --- | --- | --- |
| Model | Dimension | Eigenvalue | Condition Index | Variance Proportions | | | | |
|  |  |  |  | (Constant) | rratio1 | sratio1 | raccuracy | fratiobal |
| 1 | 1 | 3.746 | 1.000 | .00 | .00 | .00 | .00 | .00 |
|  | 2 | 1.163 | 1.794 | .00 | .01 | .02 | .00 | .00 |
|  | 3 | .075 | 7.082 | .02 | .14 | .52 | .02 | .00 |
|  | 4 | .009 | 20.376 | .49 | .74 | .45 | .70 | .04 |
|  | 5 | .007 | 22.882 | .48 | .10 | .00 | .28 | .96 |
|  |  |  |  |  |  |  |  |  |
| --- | --- | --- | --- | --- | --- | --- | --- | --- |
| a. Dependent Variable: FM | | | | | | | | |
|  |  |  |  |  |  |  |  |  |

Log

REGRESSION  
  /MISSING LISTWISE  
  /STATISTICS COEFF OUTS R ANOVA COLLIN TOL  
  /CRITERIA=PIN(.05) POUT(.10)  
  /NOORIGIN  
  /DEPENDENT FM  
  /METHOD=ENTER sratio1 raccuracy fratiobal.

Regression

Variables Entered/RemovedaVariables Entered/Removed, 表, 列标题的 1 级和行标题的 1 级, 具有 4 列和 5 行的表

|  |  |  |  |
| --- | --- | --- | --- |
| Model | Variables Entered | Variables Removed | Method |
| 1 | fratiobal, sratio1, raccuracyb | . | Enter |
|  |  |  |  |
| --- | --- | --- | --- |
| a. Dependent Variable: FM | | | |
| b. All requested variables entered. | | | |
|  |  |  |  |

Regression

Model SummaryModel Summary, 表, 列标题的 1 级和行标题的 1 级, 具有 5 列和 4 行的表

|  |  |  |  |  |
| --- | --- | --- | --- | --- |
| Model | R | R Square | Adjusted R Square | Std. Error of the Estimate |
| 1 | .953a | .909 | .841 | 7.833 |
|  |  |  |  |  |
| --- | --- | --- | --- | --- |
| a. Predictors: (Constant), fratiobal, sratio1, raccuracy | | | | |
|  |  |  |  |  |

Regression

ANOVAaANOVA, 表, 列标题的 1 级和行标题的 2 级, 具有 7 列和 7 行的表

|  |  |  |  |  |  |  |
| --- | --- | --- | --- | --- | --- | --- |
| Model | | Sum of Squares | df | Mean Square | F | Sig. |
| 1 | Regression | 2448.431 | 3 | 816.144 | 13.301 | .015b |
|  | Residual | 245.444 | 4 | 61.361 |  |  |
|  | Total | 2693.875 | 7 |  |  |  |
|  |  |  |  |  |  |  |
| --- | --- | --- | --- | --- | --- | --- |
| a. Dependent Variable: FM | | | | | | |
| b. Predictors: (Constant), fratiobal, sratio1, raccuracy | | | | | | |
|  |  |  |  |  |  |  |

Regression

CoefficientsaCoefficients, 表, 列标题的 2 级和行标题的 2 级, 具有 9 列和 8 行的表

|  |  |  |  |  |  |  |  |  |
| --- | --- | --- | --- | --- | --- | --- | --- | --- |
| Model | | Unstandardized Coefficients | | Standardized Coefficients | t | Sig. | Collinearity Statistics | |
|  |  | B | Std. Error | Beta |  |  | Tolerance | VIF |
| 1 | (Constant) | -14.218 | 15.237 |  | -.933 | .404 |  |  |
|  | sratio1 | -4.642 | 10.203 | -.099 | -.455 | .673 | .481 | 2.078 |
|  | raccuracy | 4.322 | 36.466 | .048 | .119 | .911 | .141 | 7.087 |
|  | fratiobal | 155.299 | 82.004 | .836 | 1.894 | .131 | .117 | 8.547 |
|  |  |  |  |  |  |  |  |  |
| --- | --- | --- | --- | --- | --- | --- | --- | --- |
| a. Dependent Variable: FM | | | | | | | | |
|  |  |  |  |  |  |  |  |  |

Regression

Collinearity DiagnosticsaCollinearity Diagnostics, 表, 列标题的 2 级和行标题的 2 级, 具有 8 列和 8 行的表

|  |  |  |  |  |  |  |  |
| --- | --- | --- | --- | --- | --- | --- | --- |
| Model | Dimension | Eigenvalue | Condition Index | Variance Proportions | | | |
|  |  |  |  | (Constant) | sratio1 | raccuracy | fratiobal |
| 1 | 1 | 3.221 | 1.000 | .00 | .01 | .00 | .00 |
|  | 2 | .736 | 2.092 | .00 | .32 | .00 | .00 |
|  | 3 | .035 | 9.531 | .56 | .50 | .20 | .00 |
|  | 4 | .007 | 20.958 | .44 | .17 | .79 | 1.00 |
|  |  |  |  |  |  |  |  |
| --- | --- | --- | --- | --- | --- | --- | --- |
| a. Dependent Variable: FM | | | | | | | |
|  |  |  |  |  |  |  |  |

IBM SPSS Web Report

X

The IBM SPSS Web Report is an interactive report that can be opened in a browser and contains charts, tables, and other output produced by IBM SPSS Statistics. Since this document is a single file, it can be placed on any web site without requiring special servers or setup, posted on a shared file server, copied onto portable file media, or distributed via email. Web Reports are HTML5 files that can be opened on all of the latest versions of the most commonly used browsers.

Web Report controls are disabled by the system. The browser you are using has JavaScript disabled or JavaScript is disabled by the system as a security measure. Without JavaScript, the report contains all of the same charts, tables and other objects as the full featured version, but the interactive features are not available. Check the settings for your browser to turn on JavaScript or try to open the report in a different browser. On a tablet or smart phone, you may need to install an HTML viewer application.

### Controls and Options

:   Display or hide the Edit toolbar.
:   Display the help.
:   Display the Tools menu.

### Navigation Controls

:   Open and close the navigation tree
:   Display the previous object in the document (closed objects are skipped).
:   Display the next object in the document (closed items are skipped).

### Edit Toolbar

You can use the Edit toolbar to modify the appearance of tables, including changing font attributes, background color, and number of decimal positions. You can also apply a TableLook to the entire table and transpose rows and columns. You cannot change data values in the table or edit objects other than tables.

### Tools Menu

**Print.** Prints the entire contents of the Web Report.

**Open in Simplified View.** Displays the entire contents of the Web Report in a simplified view. Instead of just displaying the select output object, all contents are displayed. You can change layers in multi-dimensional tables, but you cannot make any other changes to the table.
